# Pilot validation of the genital psoriasis reported outcomes questionnaire in an Italian cohort

**DOI:** 10.3389/fmed.2026.1815709

**Published:** 2026-07-29

**Authors:** Fabio Artosi, Elena Campione, Sara Lambiase, Chiara Cattani, Elisabetta Botti, Arianna Zangrilli, Luca Bianchi

**Affiliations:** Dermatology Unit, Department of Systems Medicine, University of Rome Tor Vergata, Rome, Italy

**Keywords:** genital, PROMS, psoriasis, quality of life, questionnaire

## Abstract

**Background:**

Genital psoriasis is a highly impactful but frequently under-recognized manifestation of psoriasis, associated with pain, burning, pruritus, and substantial impairment in sexual, relational, and emotional wellbeing. Existing patient-reported outcome measures (PROMs), such as the Genital Psoriasis Symptoms Scale (GPSS) and the Genital Psoriasis Sexual Frequency Questionnaire (GenPs-SFQ), evaluate isolated domains (physical symptoms or sexual functioning) and do not provide a multidimensional assessment of the specific burden associated with genital psoriasis. To address this gap, the Genital Psoriasis Reported Outcomes (GPRO) questionnaire was developed. This study represents the first pilot psychometric validation of the GPRO questionnaire in an Italian cohort.

**Objective:**

The study aimed to assess the feasibility, internal consistency, construct validity, structural validity, and responsiveness of the GPRO questionnaire in adult patients with genital psoriasis initiating systemic therapy.

**Methods:**

A monocentric, prospective, observational pilot study was conducted, enrolling 57 adults with clinically confirmed genital psoriasis. Assessments were performed at baseline (T0) and at week 16 (T1). Participants completed the GPRO questionnaire, GPSS, and GenPs-SFQ, and clinicians assessed disease severity using the modified version of the Physician’s Global Assessment of Genitalia (gPGA) and the Psoriasis Area and Severity Index (PASI). Analyses adhered to COnsensus-based Standards for the selection of health Measurement INstruments (COSMIN) guidance and included assessments of internal consistency (Cronbach’s *α*), exploratory factor analysis (EFA), convergent/discriminant validity (Pearson correlations), responsiveness (ΔGPRO, ΔgPGA, and anchor-based analysis), and receiver operating characteristic (ROC) curve analysis to determine the minimal important change (MIC).

**Results:**

The GPRO questionnaire showed excellent internal consistency (*α* = 0.94). The EFA supported a unidimensional structure, indicating a common latent construct that captures the global burden of genital psoriasis. At T0, the GPRO questionnaire demonstrated strong convergent validity, correlating with the gPGA (*r* = 0.77), GPSS (*r* = 0.54), and GenPs-SFQ domains (*r* = 0.7). This confirms the GPRO’s capability to integrate physical and psychosocial impacts. The questionnaire showed robust responsiveness: GPRO scores significantly decreased after treatment (*p* < 0.001), and ΔGPRO moderately correlated with ΔgPGA (*r* = 0.52). The ROC analysis demonstrated good discriminative ability for detecting clinical improvement (AUC = 0.81).

**Conclusion:**

The GPRO questionnaire demonstrated strong preliminary psychometric performance, including internal consistency, construct validity, and responsiveness. As a multidimensional PROM, it captures different aspects of genital psoriasis. These findings support the feasibility of the GPRO questionnaire and justify further evaluation in larger multicentric studies for full validation.

## 1 Introduction

Genital psoriasis is a chronic inflammatory skin condition that affects the genitalia, including the penis, scrotum, vulva, perineum, and perianal region. It is characterized by well-demarcated erythematous plaques that typically have minimal scaling due to the moist environment in these areas. Genital psoriasis is clinically significant due to its psychological impact and the unique therapeutic challenges posed by the local anatomy and humidity (1, 2).

The disease is initiated by the activation of innate immune cells (such as plasmacytoid dendritic cells, keratinocytes, and macrophages), which release cytokines that stimulate myeloid dendritic cells. These dendritic cells secrete interleukin (IL)-12 and IL-23, driving the differentiation and proliferation of T-helper (Th) cell subsets, particularly Th1, Th17, and Th22 cells (3, 4, 35).

Genital psoriasis affects approximately one-third to nearly half of all patients with psoriasis at some point during the course of the disease (5–10). Instantaneous prevalence rates from clinic-based studies range from 38 to 46% among psoriasis patients, while some population-based surveys report lifetime genital involvement in up to 63% of patients (8–10). Genital psoriasis is often under-recognized and underdiagnosed, as many patients do not discuss genital symptoms with their physicians (6, 11).

Genital psoriasis can occur at any age and is not limited to individuals with flexural psoriasis. It can be associated with more severe overall disease, an earlier age at onset, and involvement of other sites such as the scalp, nails, and inverse regions of the body (6, 8, 9).

Globally, patients with genital psoriasis experience impairment across three main domains: Physical symptoms, psychosexual and social impact, and reduced quality of daily life in carrying out normal activities. Physical symptoms include pruritus, pain, burning, dyspareunia, and, sometimes, worsening of lesions after sexual intercourse. Itching is the most prevalent symptom and can be severe, often leading to discomfort and avoidance of sexual activity (6–8). Genital psoriasis is associated with sexual dysfunction, distress, embarrassment, shame, and fear of rejection. Patients frequently report decreased sexual frequency, avoidance of intimacy, and impaired relationships (8, 10, 12, 13). Genital involvement leads to a significant impairment in overall quality of life (QoL), as measured by validated instruments such as the Dermatology Life Quality Index (DLQI). Patients face limitations in daily activities, experience social withdrawal, and exhibit increased rates of depression and anxiety. The impact on their daily functioning is disproportionate to the extent of skin involvement due to the sensitivity of the affected area (6, 8, 13–15).

Genital psoriasis can be assessed by clinicians using clinimetric scores, among which the most commonly used are the Physician’s Global Assessment of Genitalia and its modified version (gPGA) and the Psoriasis Area and Severity Index (PASI). The Physician’s Global Assessment of Genitalia is a clinician-rated scale designed specifically for genital psoriasis, emphasizing erythema and providing a reliable, standardized measure of severity in this anatomically distinct site (16). The modified version, gPGA, further refines the assessment by taking into account the characteristic prominence of erythema and minimal scaling in genital lesions, thereby improving sensitivity compared to traditional global assessments (16). The PASI remains the standard tool for evaluating overall psoriasis severity, yet it is less sensitive to small but clinically significant areas such as the genitalia and may underestimate disease burden in this region (17).

Given that genital psoriasis involves a particularly intimate and personal dimension of the patient’s experience, the assessment of disease severity cannot overlook the patient’s self-perception, as captured through patient-reported outcome measures (PROMs). Indeed, while clinician evaluation tends to be substantially objective and detached, patients with differing characteristics may report profoundly different experiences of genital psoriasis despite comparable levels of clinical severity. It is well established that factors such as female sex, the presence of a stable partner, age, disease duration, and subjective symptoms can markedly influence the personal burden of genital psoriasis (8, 11, 13, 14, 18, 19). Therefore, it is essential to complement an objective clinician-rated index with a patient-completed instrument that measures the personal activity and impact of genital psoriasis. Below is a discussion of the main PROMs currently used in the literature, specifically related to genital psoriasis.

The Dermatology Life Quality Index (DLQI) is a widely used, brief 10-item questionnaire that assesses the global impact of skin disease on patients’ lives across various domains, including symptoms, personal feelings, daily activities, leisure, work/school, relationships, and treatment. Its ease of use and broad applicability make it attractive for routine practice and research, but its generality is also its main limitation in the context of genital psoriasis: the DLQI does not assess local, genital-specific symptoms in detail, nor does it fully capture the multidimensional sexual and relational burdens that frequently accompany genital involvement. As a consequence, patients with severe genital disease may receive relatively low DLQI scores that underestimate the true burden (20, 21).

The Genital Psoriasis Symptoms Scale (GPSS) was specifically developed to quantify the psycho-physical symptom burden of genital psoriasis. The GPSS comprises eight items—itch, pain, discomfort, stinging/prickling sensation, burning, erythema, scaling, and fissuring. Each item is rated on a 0–10 scale, yielding a total score ranging between 0 and 80. This score reflects the severity of symptoms experienced over the previous 24 h. Its main strengths are symptom specificity and patient acceptability, providing clinicians with a granular picture of the current local symptom burden, which can help guide treatment decisions. However, limitations include its narrow focus on somatic symptoms: The GPSS does not assess the broader psychosocial or sexual sequelae of genital psoriasis, nor does it address the frequency or avoidance behaviors related to intimacy (7).

The Genital Psoriasis Sexual Frequency Questionnaire (GenPs-SFQ) targets sexual behavior and avoidance by asking two concise but focused questions: One assessing the frequency of sexual activity in the previous week and the other evaluating the extent to which genital psoriasis limited that activity. This simplicity allows the instrument to be sensitive to changes in sexual frequency and makes it useful as an outcome measure in intervention studies; however, its brevity is also a limitation because it cannot identify the multiple causes that reduce sexual activity (such as pain, pruritus, shame, decreased desire, and partner-related issues). Additionally, it does not measure broader dimensions of sexual function or distress (8, 10).

Several instruments focus specifically on sexual function or sexual quality of life (QoL) but are not specific to genital psoriasis. The Sexual Quality of Life Questionnaire for Men (SQoL-M), the International Index of Erectile Function (IIEF), the Female Sexual Distress Scale (FSDS), and the Female Sexual Function Index (FSFI) are psychometrically robust tools for assessing global male sexual QoL, multiple domains of male sexual function, female sexual distress, and female sexual function across domains (desire, arousal, lubrication, orgasm, satisfaction, and pain), respectively. The added value of these instruments in genital psoriasis research lies in their ability to quantify sexual dysfunction or distress. Their limitation is conceptual, as these scales do not capture dermatological symptoms or the specific interplay between visible genital lesions, stigma, and sexual behavior that is typical of genital psoriasis (12, 13, 22, 23).

The Skin-Related Sexual Life Questionnaire (SRSLQ) and tools such as Qualipsosex were developed to examine the psychosexual and quality-of-life consequences of skin disease in a broader context (24). These instruments provide important psychosocial insights that are lacking in the GPSS and GenPs-SFQ. However, they do not specifically focus on genital-site symptoms and may therefore miss certain acute local physical drivers of impairment (13, 25).

The Self-Administered Psoriasis Area and Severity Index (SAPASI) allows patients to self-assess erythema, induration, and scaling across different body regions using a body silhouette. While the SAPASI captures the overall cutaneous severity from the patient’s perspective, it tends to underestimate the involvement of small but high-impact sites such as the genitals because area-based instruments allocate a low surface area to the genital region despite the disproportionate QoL impact (26).

Finally, disease-specific instruments for vulvar diseases such as the Vulvar Quality of Life Index (VQLI) provide a focused assessment of vulvar symptoms and their QoL consequences. However, the scope of the VQLI is anatomical rather than disease-specific, and it may not translate directly to male genital disease or psoriatic-specific pathophysiology (27).

Overall, the limitations of the available instruments converge on two issues: (1) many standard tools are not sufficiently sensitive to the disproportionate burden of small, high-impact sites such as the genitals, and (2) no single existing instrument integrates local somatic symptoms, psychosexual distress, avoidance behaviors, and functional daily-life consequences into a unified, genital-specific PROM. This gap motivated the development of the Genital Psoriasis Reported Outcomes (GPRO) questionnaire.

The reference point for drafting PROMs is the COnsensus-based Standards for the selection of health Measurement INstruments (COSMIN), an internationally developed methodological framework designed to improve the selection, development, and evaluation of PROMs and other health measurement instruments (28).

The GPRO questionnaire is a PROM that addresses the three main symptom domains reported by patients with genital psoriasis, as indicated in the literature. We, therefore, conducted a prospective, interventional pilot study at the Dermatology Unit of “Tor Vergata” University Hospital in Rome to evaluate the GPRO’s capability in reflecting the severity of genital psoriasis and monitoring its changes over time.

## 2 Methods

### 2.1 Study design

This was a prospective, observational, single-center study designed to evaluate the psychometric properties, clinical validity, and responsiveness of the newly developed Genital Psoriasis Reported Outcome (GPRO) questionnaire in adult patients with genital psoriasis. Participants were evaluated at baseline (T0), corresponding to the initiation of a new systemic therapy, and after 16 ± 1 weeks (T1), a time window selected based on the expected therapeutic response according to current dermatological guidelines.

The study allowed assessment of (i) internal consistency, (ii) structural validity through exploratory factor analysis (EFA), (iii) construct validity by correlating the GPRO questionnaire with validated genital psoriasis PROMs (the GPSS and GenPs-SFQ), (iv) criterion validity versus clinician-rated genital psoriasis severity (gPGA), (v) anatomical specificity relative to global skin severity (PASI), and (vi) responsiveness to clinical change using anchor- and distribution-based methods.

The study protocol was approved by the local ethics committee (Comitato Etico Territoriale Lazio Area 2 – CET2PTV) and conducted in accordance with the Declaration of Helsinki.

### 2.2 Participants

A total of 57 consecutive patients attending the Dermatology Clinic of Policlinico Tor Vergata (Rome, Italy) were enrolled. All patients provided written informed consent prior to participation.

#### 2.2.1 Inclusion criteria

The inclusion criteria were as follows:

1. Ability to comprehend study procedures and provide written informed consent.
2. Age ≥18 years.
3. Clinical diagnosis of genital psoriasis, with or without involvement of other body areas.
4. Initiation of a new systemic therapy for psoriasis at enrollment, according to regional and national guidelines.

#### 2.2.2 Exclusion criteria

The exclusion criteria were as follows:

1. Other dermatologic conditions of the genital area (e.g., eczema, atopic dermatitis) or open wounds/scarring interfering with assessment.
2. Autoimmune diseases other than psoriasis.
3. Congenital or acquired genital abnormalities interfering with scoring.
4. Use of systemic or topical corticosteroids, topical calcineurin inhibitors, or vitamin D analogs within the previous 4 weeks.
5. Ongoing or recent (<4 weeks) systemic therapies approved for psoriasis.
6. Pregnancy.
7. Active infection.
8. Inability to comply with study visits or instructions.

### 2.3 GPRO questionnaire

The GPRO questionnaire comprises two sections. The first collects demographic and clinical information. The second includes eight items assessing physical, psychological, and functional impairment related to genital psoriasis. Items are scored using Likert-type response options with heterogeneous ranges (0–3 for items 1, 2, 5, and 6; 0–4 for items 4, 7, and 8; 0–2.5 for item 3). Item 3 is completed only if item 2 is scored ≥1. The total score ranges from 0 to 26.5, with higher scores reflecting greater impact. The complete GPRO questionnaire can be found in the Supplementary material section.

#### 2.3.1 Development of the GPRO questionnaire

The development process followed COSMIN guidelines (28).

Phase 1—Construct definition and item generation: Items were derived from a literature review of existing PROMs for genital psoriasis (the GPSS, GenPs-SFQ, and DLQI), expert clinical experience, and semi-structured patient interviews exploring relevant domains such as pain, discomfort, shame, sexual avoidance, and behavioral adaptations.

Phase 2—Content validity: A multidisciplinary expert panel assessed clarity and relevance. Cognitive debriefing in a small patient sample was used to refine item wording, the reference timeframe (“past 15 days”), and response formats.

Phase 3—Psychometric validation: The finalized eight-item version underwent quantitative evaluation of internal consistency, structural validity, convergent and criterion validity (vs. the GPSS, GenPs-SFQ, gPGA, and PASI), and responsiveness to treatment.

### 2.4 Comparator instruments

Participants also completed the GPSS (eight symptoms scored 0–10) and the GenPs-SFQ. The GPSS is described as a patient-reported outcome measure specifically developed for genital psoriasis. Its structure consists of individual items assessing key symptoms identified as relevant through literature review and patient interviews: Itch, pain, scaling, redness/erythema, and stinging/burning. Each symptom is rated by the patient using a numeric rating scale, typically ranging from 0 (no symptom) to 10 (worst imaginable severity), with total scores ranging from 0 to 80 (7).

The GenPs-SFQ is a patient-reported outcome measure specifically developed to assess the impact of genital psoriasis on sexual activity frequency. Its structure consists of two items that directly ask patients about limitations in sexual activity frequency due to genital psoriasis. The total score for the GenPs-SFQ is calculated by summing the responses to its two items, which assess the frequency of sexual activity and the degree to which genital psoriasis limits sexual activity frequency (8).

### 2.5 Clinical assessments

A dermatologist evaluated genital psoriasis severity using the 5-point modified Genital Physician Global Assessment (gPGA, 0–4). Overall disease severity was assessed using the PASI.

The gPGA scale is based on a static assessment of the clinical appearance of genital lesions, considering parameters such as erythema, induration, and scaling, with particular attention to the intensity of erythema, which often predominates at this site. The score is assigned using an ordinal scale (e.g., 0 = clear, 1 = almost clear, 2 = mild, 3 = moderate, and 4 = severe), facilitating the classification of severity and monitoring of therapeutic response over time.

PASI scores quantify the severity of psoriasis by evaluating both the extent of body surface area involvement and the intensity of key clinical features: Erythema (redness), induration (thickness), and scaling (desquamation). The body is divided into four regions: Head, trunk, upper extremities, and lower extremities. For each region, the percentage of the affected area is scored from 0 to 6, and the severity of each clinical feature is scored from 0 (none) to 4 (maximum). The resulting scores are then weighted by the proportion of total body surface area represented by each region and summed to yield a total PASI score ranging from 0 (no disease) to 72 (maximal disease severity).

### 2.6 Study procedures

At both T0 and T1, patients completed the GPRO questionnaire, GPSS, and GenPs-SFQ before the clinical evaluation. Data were anonymized and stored in a dedicated database. Questionnaires with >10% missing items were excluded; for ≤10% missingness, single-item mean imputation was applied.

### 2.7 Statistical analysis

Analyses were performed in Python (v3.11). Descriptive statistics included means and standard deviations or medians and interquartile ranges for continuous variables and frequencies for categorical variables. Internal consistency was assessed using Cronbach’s *α*, corrected item–total correlations, inter-item correlations, and α if item deleted (29).

#### 2.7.1 Construct and all criterion validity

Pearson correlations were computed between the GPRO questionnaire and GPSS, GenPs-SFQ, and gPGA at T0. Correlation strength was interpreted as follows: negligible (<0.20), weak (0.20–0.39), moderate (0.40–0.59), strong (0.60–0.79), and very strong (≥0.80) (30). Linear regression models were fitted separately for each PROM with the gPGA as the dependent variable (primary outcome) and PASI as a secondary outcome (30).

#### 2.7.2 Responsiveness

Change scores (ΔGPRO, ΔgPGA) were computed. Anchor-based responsiveness used ΔgPGA as the external criterion (responder = improvement ≥1 point). Analyses included Pearson correlations between ΔGPRO and ΔgPGA, receiver operating characteristic (ROC) curves with AUC and Youden index-derived minimal important change (MIC), and distribution-based indices (SRM, effect size, Cohen’s dz., and SEM-based MIC) (31).

#### 2.7.3 Structural validity

##### 2.7.3.1 Exploratory factor analysis

An exploratory factor analysis (EFA) was conducted on the eight items of the GPRO questionnaire at baseline (T0) to examine its underlying factor structure (32, 33). Data from 57 patients were included, using item-level scores from GPRO T0 Q1 to Q8.

Given the absence of a previously validated structural model, the analysis was conducted within an exploratory framework. However, interpretation of the factor solution was guided by an *a priori* theoretical model encompassing three conceptual domains: Physical symptomatology (Q2–Q4), psychosexual/relational distress (Q1, Q5, Q6), and impairment in daily activities (Q7–Q8). These domains were used to support clinical interpretation of the extracted factors rather than to impose a predefined structure on the model.

Factor extraction was performed using scikit-learn’s FactorAnalysis algorithm. The number of factors to retain was evaluated based on multiple criteria, including the Kaiser criterion (eigenvalue > 1), inspection of the scree plot, and the interpretability of the factor solution. To further explore the theoretical structure, a three-factor solution was examined *post hoc* and compared with the empirically driven factor pattern (34).

Factor loadings were examined to identify the strongest associations between items and latent dimensions. Communalities were calculated as the sum of squared loadings, and the item-specific contribution within the factor model was assessed accordingly. Loadings ≥0.40 were considered indicative of a meaningful association with a factor, and cross-loadings were evaluated. Communalities >0.40 were interpreted as evidence of adequate representation of the item within the factor model.

A confirmatory factor analysis (CFA) was not performed in the present study due to the limited sample size, which was not sufficient to support stable estimation of multi-factor measurement models. In accordance with psychometric validation frameworks, EFA was, therefore, considered the most appropriate initial step to explore the latent structure of the instrument. Future studies with larger and independent samples will be required to formally test the hypothesized factor structure through CFA and to assess its generalizability.

#### 2.7.4 Floor and ceiling effects

The proportion of respondents scoring minimum or maximum values for each item was calculated; values <15% were considered acceptable (28).

## 3 Results

A total of 57 patients with clinically confirmed genital psoriasis were enrolled in the study, with an almost equal sex distribution (50.9% female and 49.1% male). The mean age of the cohort was 49.65 years (SD 14.60; median 49; IQR 38–60), and the mean BMI was 25.13 kg/m^2^ (SD 4.54). All patients initiated a new systemic therapy at baseline (T0), reflecting the heterogeneity of treatment approaches observed in clinical practice. The most frequently prescribed drugs included tildrakizumab 100 mg, apremilast, and adalimumab, while other biologic or small-molecule agents were used less commonly (Table 1).

**Table 1:** Systemic drugs prescribed for enrolled patients.

Drug	Number of patients
Tildrakizumab 100 mg	12
Apremilast	10
Adalimumab	7
Tildrakizumab 200 mg	6
Bimekizumab	5
Brodalumab	4
Ixekizumab	4
Deucravacitinib	3
Certolizumab Pegol	2
Risankizumab	1
Ustekinumab	1
Dimetil fumarato	1
Metotrexato	1

At T0, the descriptive analysis of all patient-reported and clinician-reported measures indicated a substantial burden of disease. The GPRO total score had a mean of 18.7 (SD 12.8; median 14; range 1–46), corresponding to elevated levels of impairment across physical, psychological, and relational domains. After 16 weeks of systemic therapy (T1), the mean GPRO score decreased to 5.3 (SD 6.9; median 3), indicating a marked reduction in patient-reported burden. A similar pattern was observed for the GPSS, which declined from a mean of 22.1 (SD 15.1) at T0 to 6.4 (SD 7.6) at T1, indicating a substantial decrease in reported physical genital symptoms, including pruritus, pain, and burning. Improvements in sexual functioning were also observed. In the GenPs-SFQ, both the frequency of sexual activity and the extent of avoidance behavior decreased between T0 and T1 in intimacy-related behaviors. Clinician-assessed measures showed that the gPGA decreased from 2.9 (SD 0.9) at baseline to 1.4 (SD 0.9) at follow-up, while the PASI declined from 8.3 (SD 8.0) to 2.4 (SD 2.9), reflecting a reduction in clinician-rated disease severity over time. All values for the clinimetric scores and questionnaires are reported in Table 2.

**Table 2:** Descriptive statistics of all clinical and patient-reported outcomes at baseline (T0) and week 16 (T1).

Outcome	Timepoint	Mean	SD	Median
GPRO (total score)	T0	18.68	12.78	14
	T1	5.25	6.90	3
GPSS	T0	22.05	15.08	20
	T1	6.37	7.56	3
GenPs-SFQ part 1 (sexual frequency)	T0	8.26	6.88	6
	T1	3.98	5.25	2
GenPs-SFQ part 2 (avoidance)	T0	15.82	8.55	17
	T1	6.00	6.86	4
gPGA	T0	2.93	0.90	3
	T1	1.44	0.86	1
PASI	T0	8.25	8.02	6.15
	T1	2.37	2.94	1.10

SD, standard deviation.

**Figure 1:**
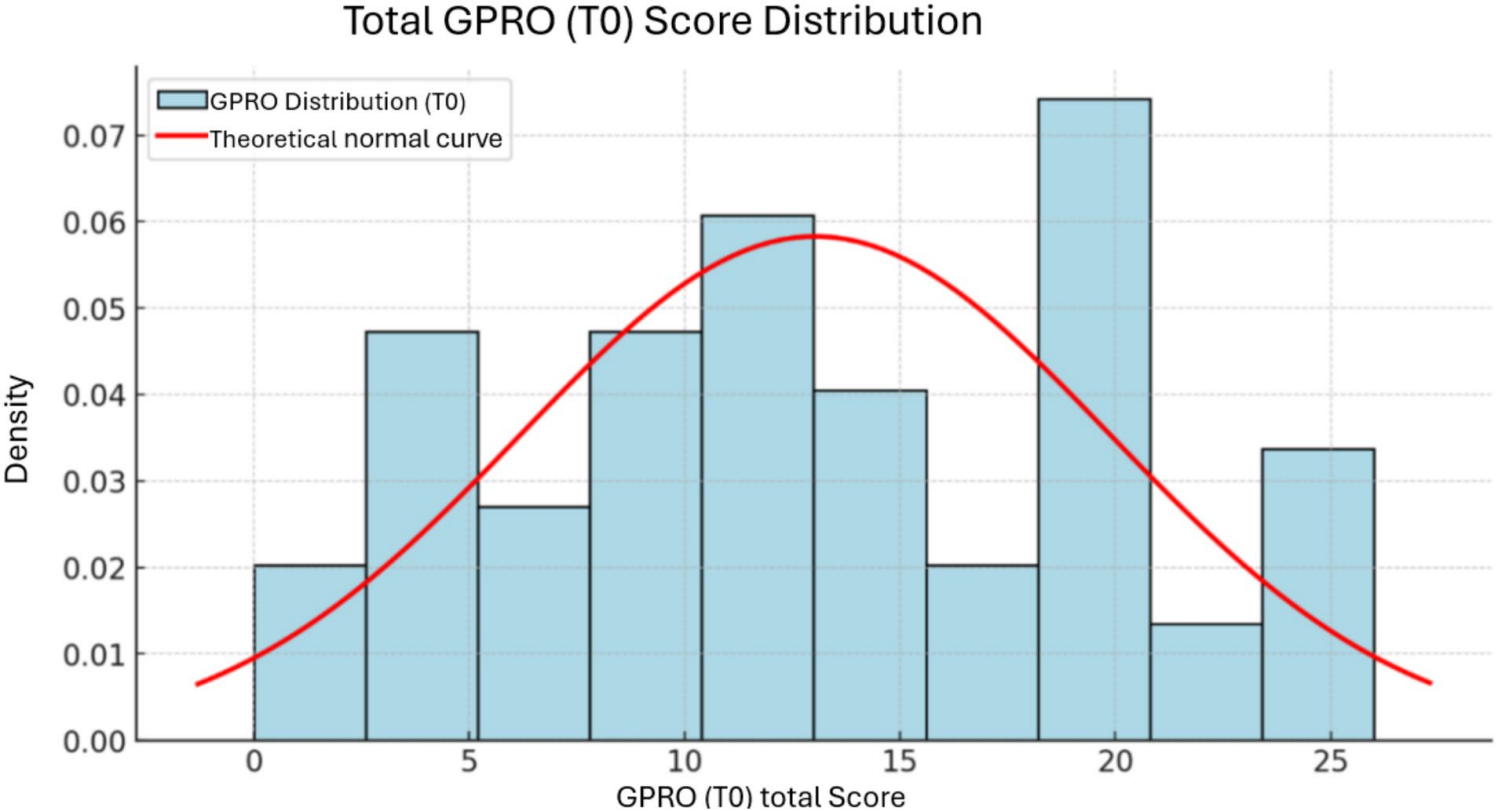
The histogram shows the distribution of total scores obtained by patients at baseline. The superimposed density curve indicates an approximately normal trend, confirmed by the Shapiro–Wilk test (*p* = 0.112). The variability of scores reflects wide heterogeneity in the subjective perception of genital discomfort among patients before starting systemic treatment.

An analysis of the distributional properties of the GPRO questionnaire further characterized these changes. At T0, GPRO total scores followed an approximately normal distribution (Shapiro–Wilk *W* = 0.966, *p* = 0.112), indicating adequate variability without substantial skewness. At T1, the distribution deviated significantly from normality (*W* = 0.850, *p* < 0.001), with a clustering of scores toward the lower end of the scale. This pattern reflects a shift toward lower values at follow-up (Figure 1).

**Figure 2:**
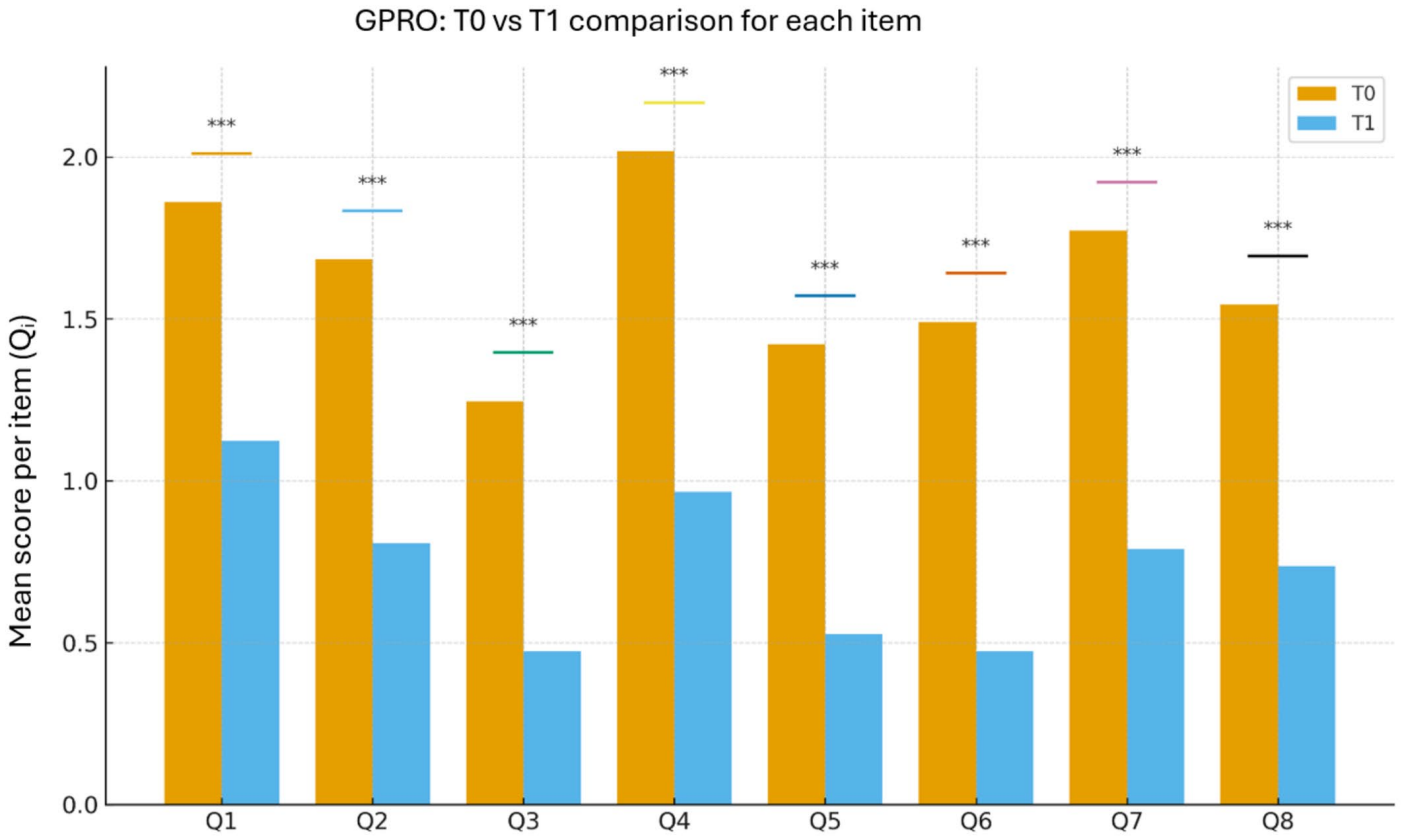
Comparison of mean scores for individual GPRO questionnaire items between T0 and T1. Mean values (± standard deviation) for the eight questions (Q1–Q8) showed a significant reduction between T0 (baseline) and T1 (follow-up). All items showed a statistically significant improvement (*p* < 0.001 for Q1, Q2, Q4, Q5, Q6, Q7, and Q8; *p* < 0.01 for Q3), indicating a decrease in patients’ perceived discomfort after treatment. Error bars represent the standard deviation.

A domain-level analysis of the GPRO questionnaire showed that all eight items exhibited statistically significant improvements between baseline and follow-up. Paired analyses demonstrated highly significant reductions for seven items (*p* < 0.001) and a significant reduction for the remaining item (*p* < 0.01). The largest absolute changes were observed in items related to physical discomfort, emotional disturbance, and relational difficulties, indicating consistent improvements across multiple domains assessed by the instrument. The uniform direction of change across items is consistent with the concept of the GPRO questionnaire reflecting a coherent underlying construct (Figure 2).

### 3.1 Linear regression analysis

**Figure 3:**
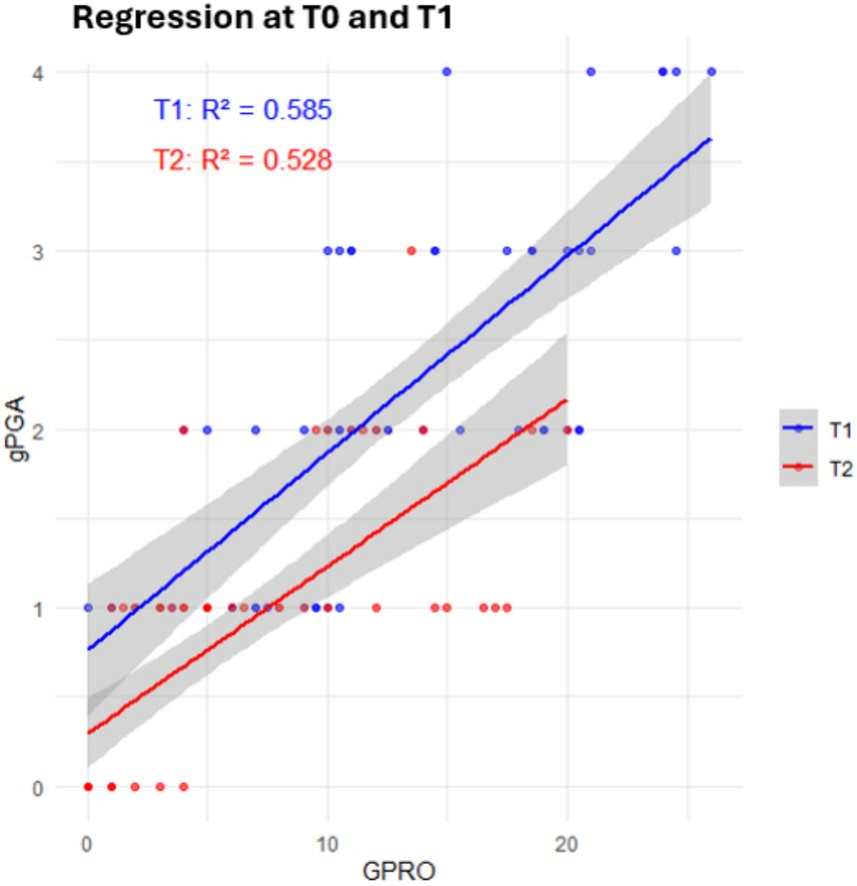
Linear regression between the gPGA and GPRO questionnaire at T0 and T1. The GPRO questionnaire showed the strongest correlation with genital clinical severity at both time points, with a clear reduction in severity at T1.

**Figure 4:**
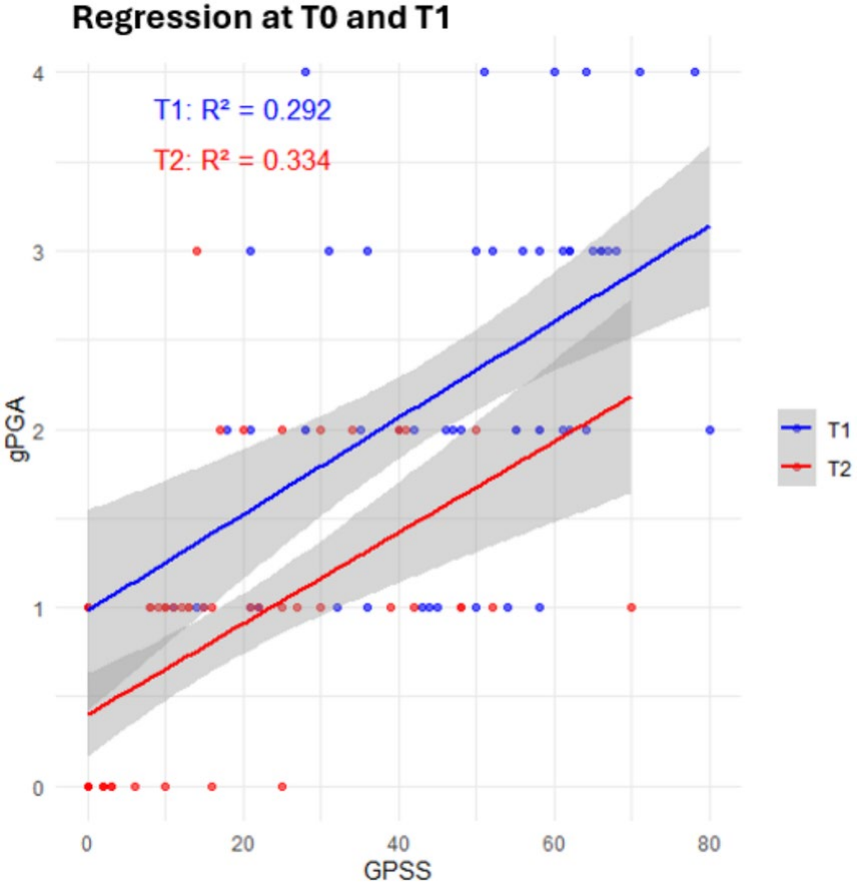
Linear regression between the gPGA and GPSS at T0 and T1. The correlation was moderate and increased slightly at T1, suggesting a relationship between residual physical symptoms and clinical severity.

**Figure 5:**
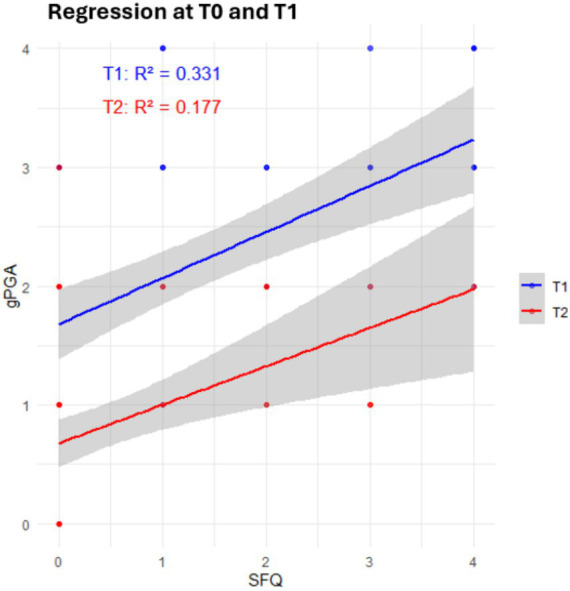
Linear regression between the gPGA and SFQ at T0 and T1. The correlation decreased significantly after treatment, indicating psychosexual improvement independent of clinical severity.

**Figure 6:**
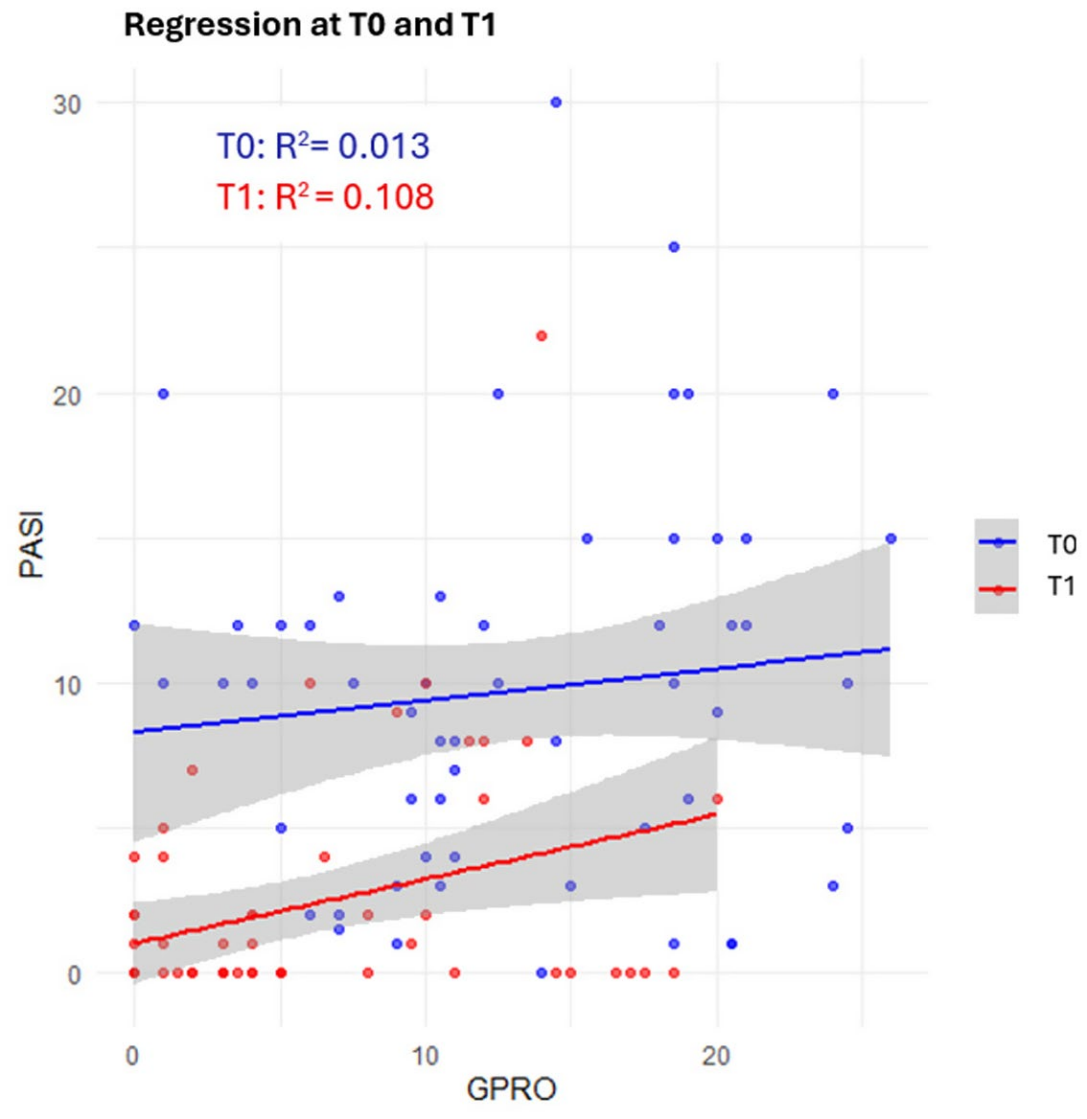
Linear regression between the PASI and GPRO questionnaire at T0 and T1.

To further evaluate the relationship between subjective and objective assessments, linear regression models were constructed separately for each patient-reported measure at both time points. The GPRO questionnaire demonstrated the strongest association with clinical severity, explaining 58.5% of the variance in the gPGA at T0 and 52.8% at T1 (both *p* < 0.001, Figure 3). This strong association reflects close alignment between GPRO scores and clinician-assessed disease severity. In contrast, the GPSS showed more moderate associations with the gPGA (*R*^2^ = 0.292 at T0 and 0.334 at T1, Figure 4), indicating a weaker relationship with clinician-rated severity compared to the GPRO questionnaire. The association between the SFQ and gPGA decreased from T0 to T1 (Figure 5), showing reduced correspondence between sexual avoidance measures and clinician-rated severity at follow-up. None of the patient-reported measures showed meaningful associations with the PASI at either time point (R^2^ consistently <0.05; Figure 6), indicating limited correspondence between genital-specific patient-reported outcomes and overall body surface disease severity.

### 3.2 Internal consistency analysis

**Figure 7:**
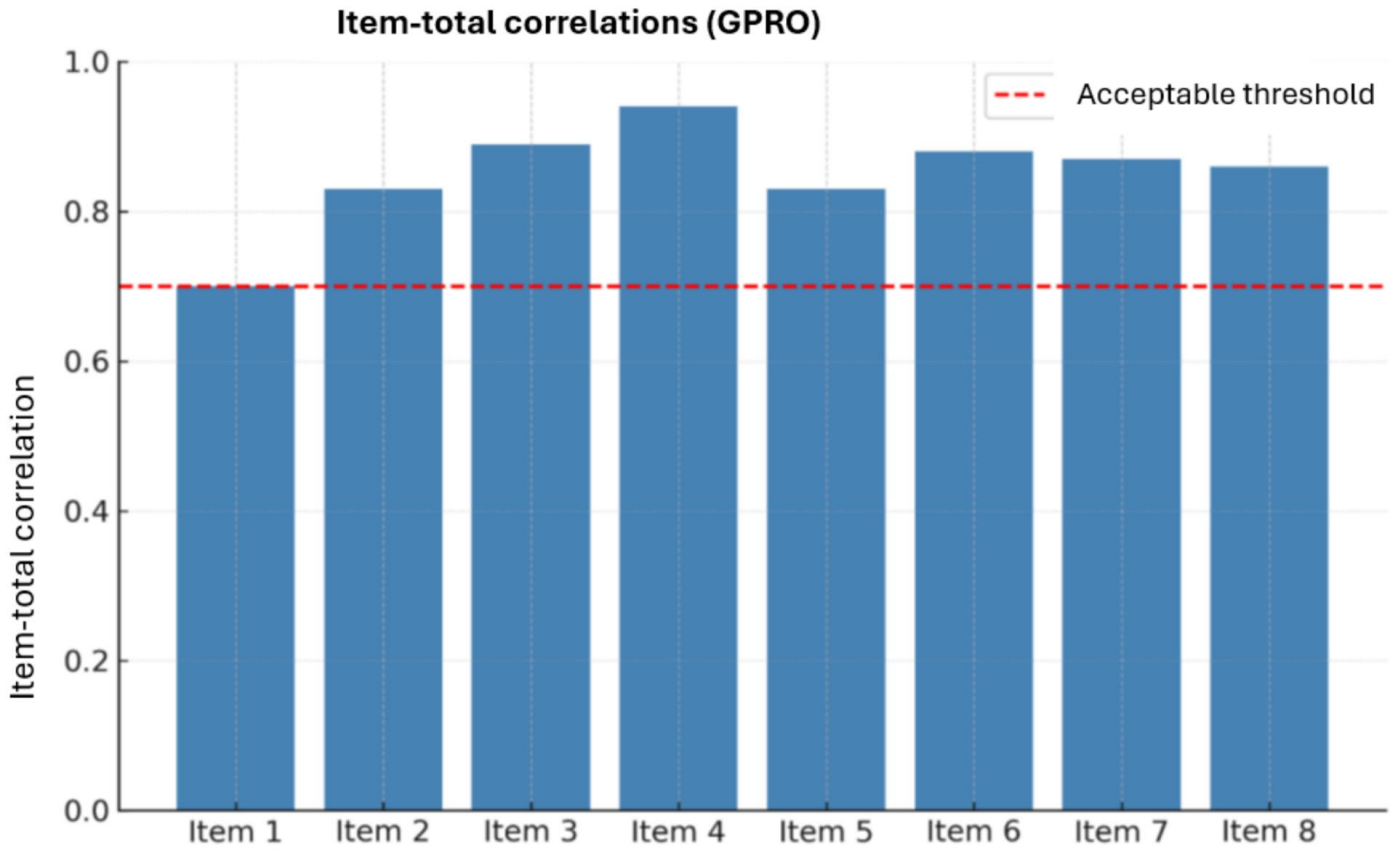
Corrected item–total correlations for the GPRO questionnaire. The bars represent the corrected item–total correlation coefficients for each item of the GPRO questionnaire, reflecting the contribution of each item to the overall internal consistency of the scale. The red dashed line (threshold value = 0.70) indicates the commonly accepted cutoff for an adequate item–total correlation. All items exceeded this threshold, demonstrating excellent internal homogeneity and indicating that no item showed weak or inconsistent alignment with the underlying construct being measured. The overall Cronbach’s alpha coefficient was 0.94.

**Figure 8:**
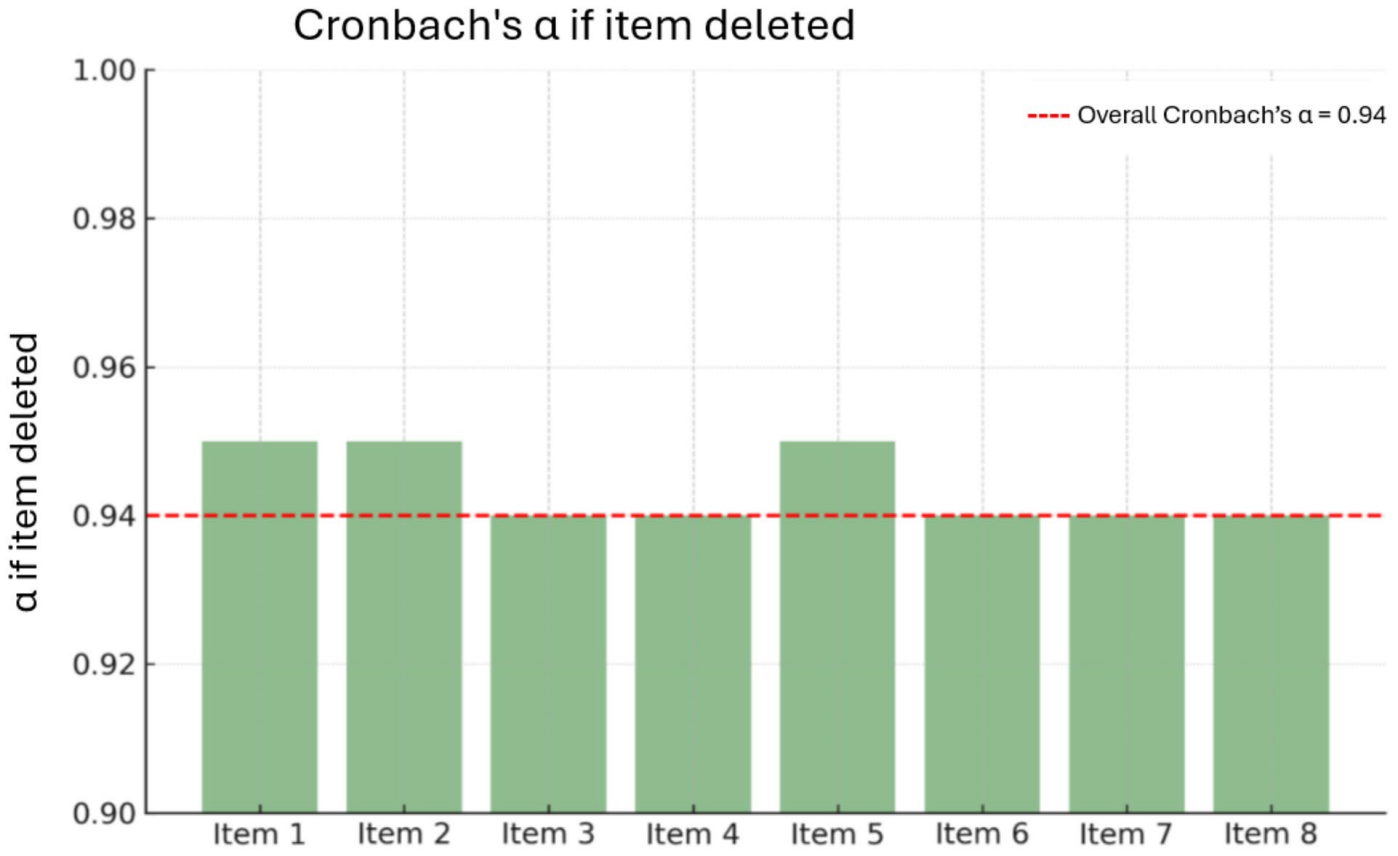
Cronbach’s *α* coefficient when individual items were removed (GPRO). The graph shows the change in Cronbach’s α coefficient when each GPRO item was removed. The dashed red line represents the overall α value obtained for the complete scale (*α* = 0.94). The absence of significant increases in the coefficient above this line indicates that no item, if eliminated, would improve the overall reliability of the instrument. This result confirms the psychometric soundness and internal consistency of the entire GPRO scale.

Internal consistency analyses demonstrated excellent reliability of the GPRO questionnaire, with Cronbach’s *α* = 0.94 (95% CI 0.91–0.96). Item–total correlations exceeded 0.70 for all items, and the mean inter-item correlation was 0.71, indicating a high degree of internal homogeneity (Figure 7). Conditional reliability analyses showed that deleting any single item would not increase Cronbach’s α (range 0.92–0.95), indicating that all items contribute meaningfully to the overall scale without evidence of excessive redundancy (Figure 8).

#### 3.2.1 Responsiveness

**Figure 9:**
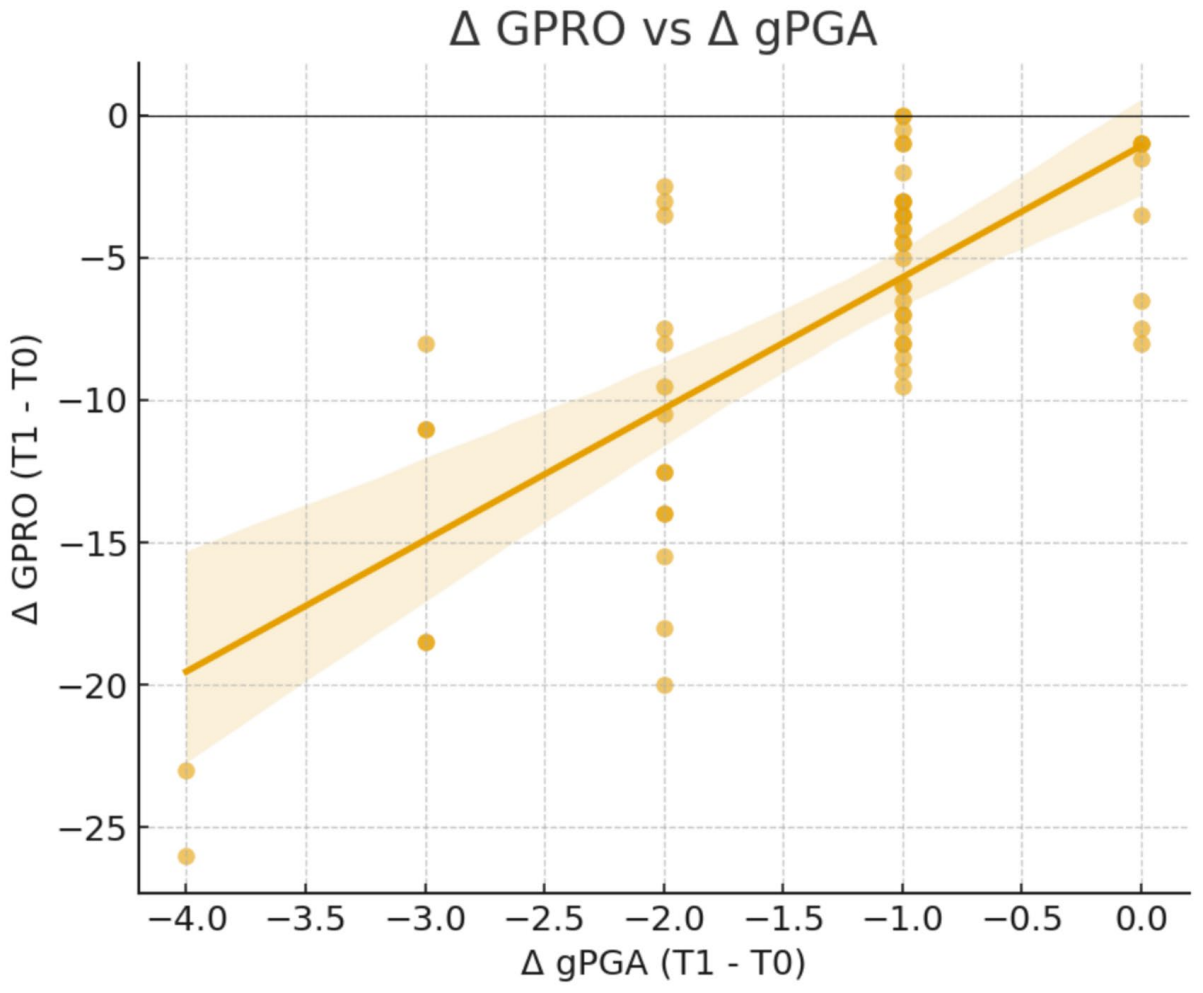
Relationship between the change in GPRO scores (ΔGPRO) and the change in clinical severity (ΔgPGA) between T0 and T1. A moderate to strong and statistically significant correlation was observed (*r* = 0.52; *p* = 0.003), with reductions in GPRO scores proportional to clinical improvement assessed by the gPGA.

**Figure 10:**
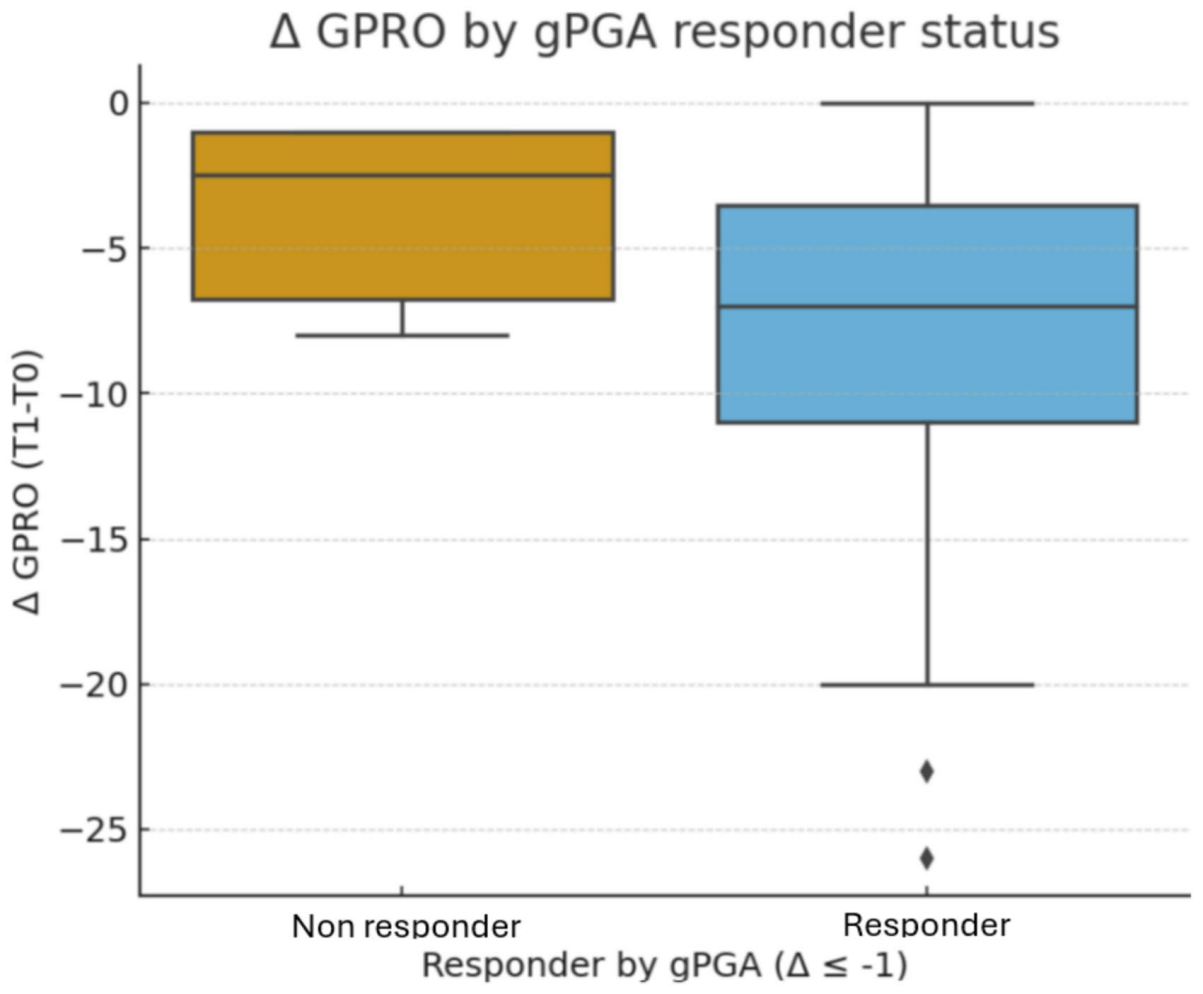
Distribution of changes in the total GPRO score (ΔGPRO) in responders and non-responders according to the clinical change in the gPGA (responders: ΔgPGA ≤ − 1). Responders showed a significantly greater reduction in GPRO scores than non-responders (*p* < 0.001), confirming the questionnaire’s ability to detect perceived clinical change.

To assess responsiveness, the change in GPRO scores between T0 and T1 (ΔGPRO) was examined in relation to the clinician-rated change in the gPGA (ΔgPGA). The correlation between these change scores was moderate to strong (*r* = 0.52, *p* = 0.003), indicating an association between changes in patient-reported burden and changes in clinician-assessed severity (Figure 9). When participants were stratified according to the anchor-based definition of clinical response (responder = ΔgPGA ≤ − 1), responders exhibited larger reductions in GPRO scores compared to non-responders, with a highly significant difference between the groups (*p* < 0.001; Figure 10).

**Figure 11:**
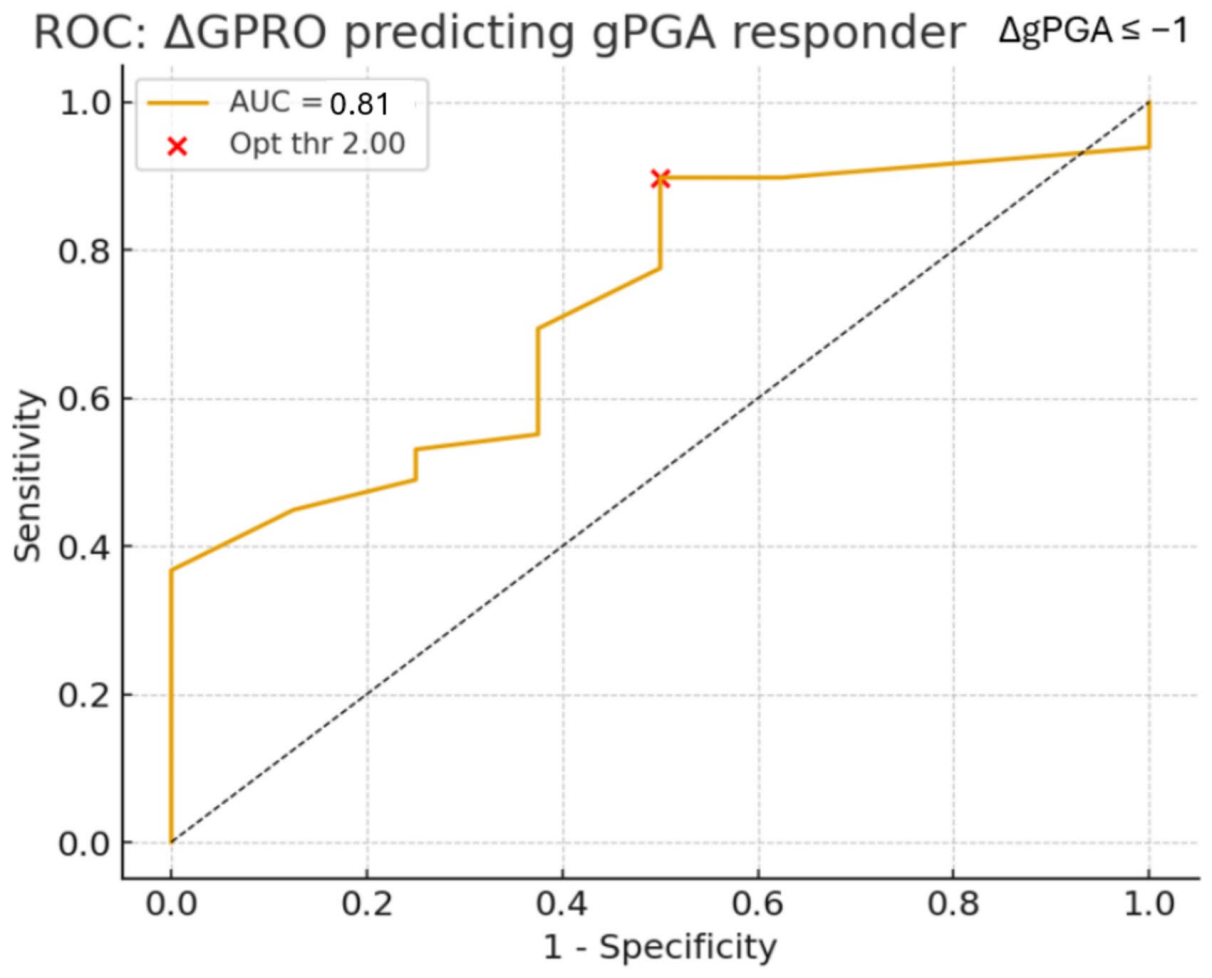
Receiver operating characteristic curve of the change in GPRO scores (ΔGPRO) for discriminating responders (ΔgPGA ≤ − 1). The AUC was 0.81 (95% CI: 0.72–0.90), indicating good discriminatory capacity. The optimal threshold identified using the Youden index was −1.50 points, corresponding to the anchor-based MIC.

Receiver operating characteristic analysis further evaluated the discriminative capacity of the GPRO questionnaire. The AUC for ΔGPRO in identifying gPGA-defined responders was 0.81 (95% CI 0.72–0.90), indicating good discrimination according to COSMIN criteria (Figure 11). The optimal cut-off for defining the MIC was −1.50 points, as determined by the Youden index. This value corresponds to the threshold that best differentiates responders from non-responders in this cohort. Clinically, a reduction of at least 1.5 points may represent the smallest change associated with a clinically meaningful improvement in genital psoriasis-related symptoms or burden.

### 3.3 Analysis of the internal structure and construct validity of the GPRO questionnaire

**Figure 12:**
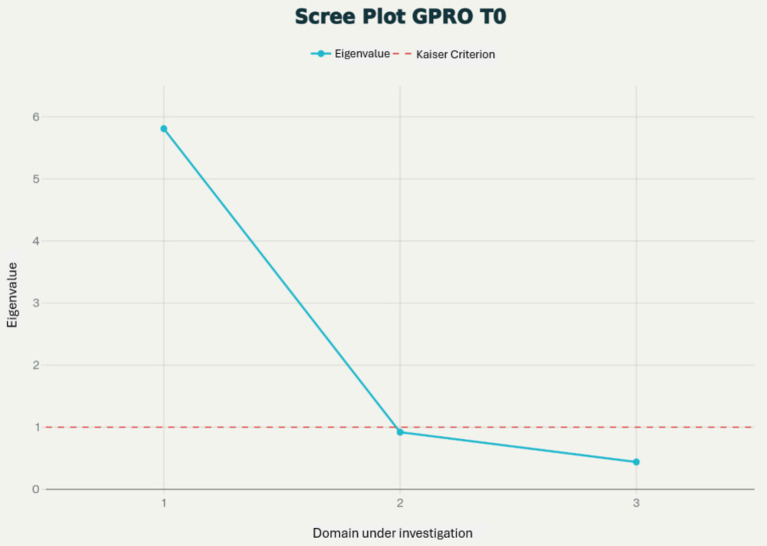
Scree plot of the GPRO questionnaire at baseline (T0). The plot shows the eigenvalues of the extracted components, with a marked drop after the first component. Only the first component exceeds the Kaiser criterion (eigenvalue > 1), suggesting a dominant underlying dimension and limited evidence for additional distinct factors..

Exploratory factor analysis revealed a dominant single-factor structure for the GPRO questionnaire at T0, with Factor 1 explaining 81.0% of the total variance (eigenvalue = 5.81), far exceeding the Kaiser criterion (Figure 12). The factor solution suggested a dominant general dimension, with the first factor showing the strongest contribution and the remaining factors contributing substantially less to the common variance structure. All eight items showed strong loadings on Factor 1 (range: 0.69–0.99), with Q7 exhibiting the highest loading (0.99) and Q3 the lowest (0.69) (Figure 12). Loadings on Factors 2 and 3 were minimal (mostly | < 0.40|), indicating limited additional structure beyond the dominant factor. Communalities were high across items (range: 0.52–1.19; mean = 0.86), indicating good representation of items within the factor solution (Figure 12). These findings are consistent with a predominantly unidimensional structure of the GPRO total score while maintaining the conceptual framework.

**Figure 13:**
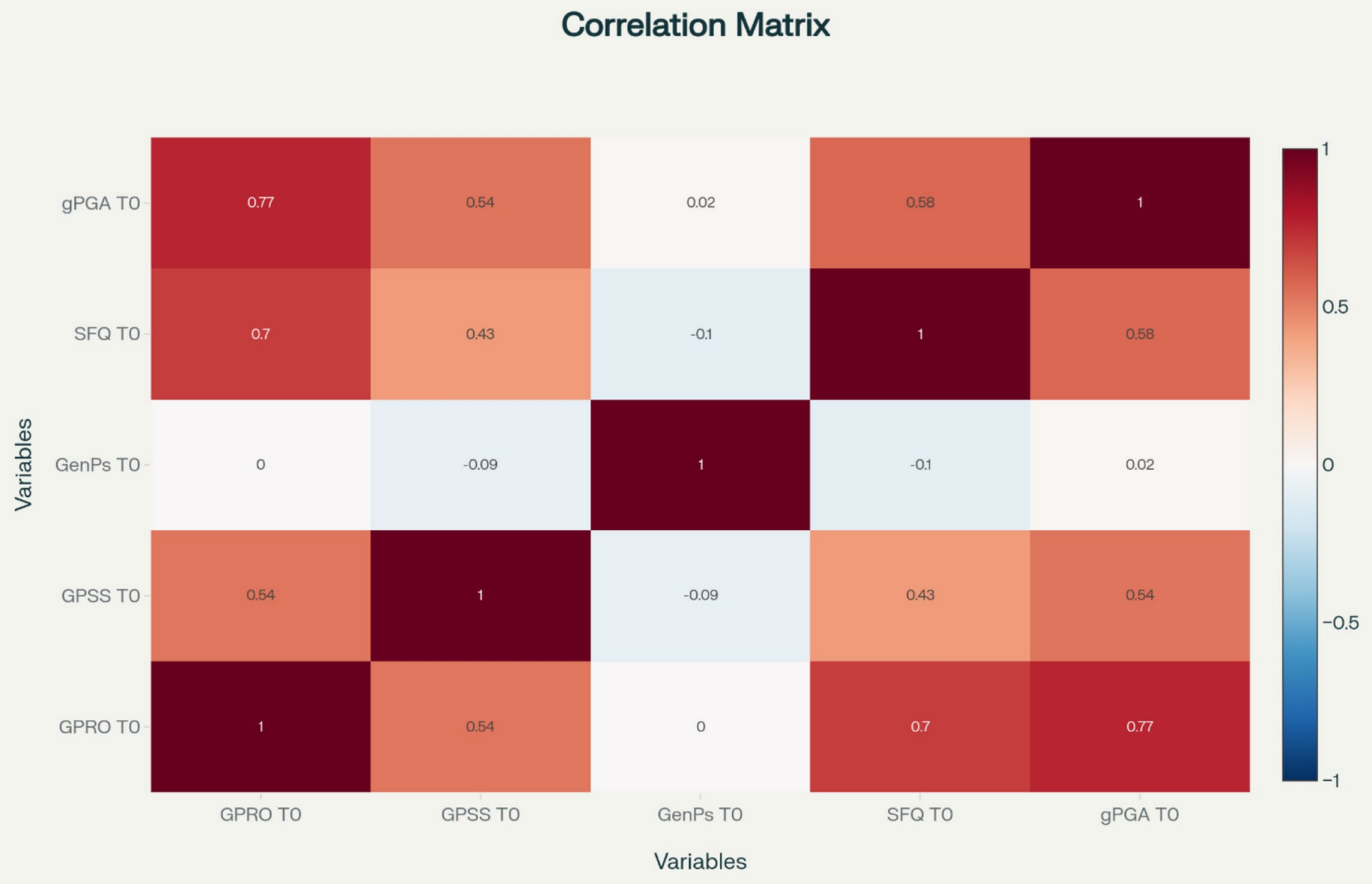
Pearson correlation coefficients between the GPRO total score and the other questionnaires administered (the GPSS, gPGA, and GenPs-SFQ Part 1 and Part 2) at baseline (T0). Higher correlation values (in red) indicate a stronger positive linear association between the instruments. A strong correlation was evident between the GPRO questionnaire, gPGA, and SFQ Part 2, consistent with convergent validity for the clinical severity and relational impact domains.

Convergent validity analyses showed strong correlations between the GPRO questionnaire and both GPSS and SFQ avoidance scores at baseline, as well as a very strong correlation with the gPGA (*r* = 0.77, *p* < 0.001). In contrast, no correlation was observed with the GenPs sexual frequency item (*r* = 0.00, *p* = 0.978), indicating a lack of association with this specific behavioral measure (Table 3; Figure 13). These results show differential relationships across related constructs.

**Table 3:** Pearson correlation coefficients between the total GPRO score and established clinical and patient-reported outcome measures assessing genital psoriasis severity, symptom burden, and psychosexual impact.

Variable	Pearson *r* with GPRO	*p*-value	Interpretation
GPSS (T0)	0.54	<0.001	Moderate positive correlation
gPGA (T0)	0.77	<0.001	Strong positive correlation
GenPs frequency (T0)	0.00	0.978	No correlation
SFQ avoidance (T0)	0.70	<0.001	Strong positive correlation

Strong correlations were observed with gPGA and SFQ avoidance scores, while a moderate correlation was found with the GPSS. No significant correlation emerged with GenPs frequency scores, supporting the discriminant specificity of the GPRO construct. Correlation strength was interpreted as negligible (<0.20), weak (0.20–0.39), moderate (0.40–0.59), strong (0.60–0.79), and very strong (≥0.80).

**Figure 14:**
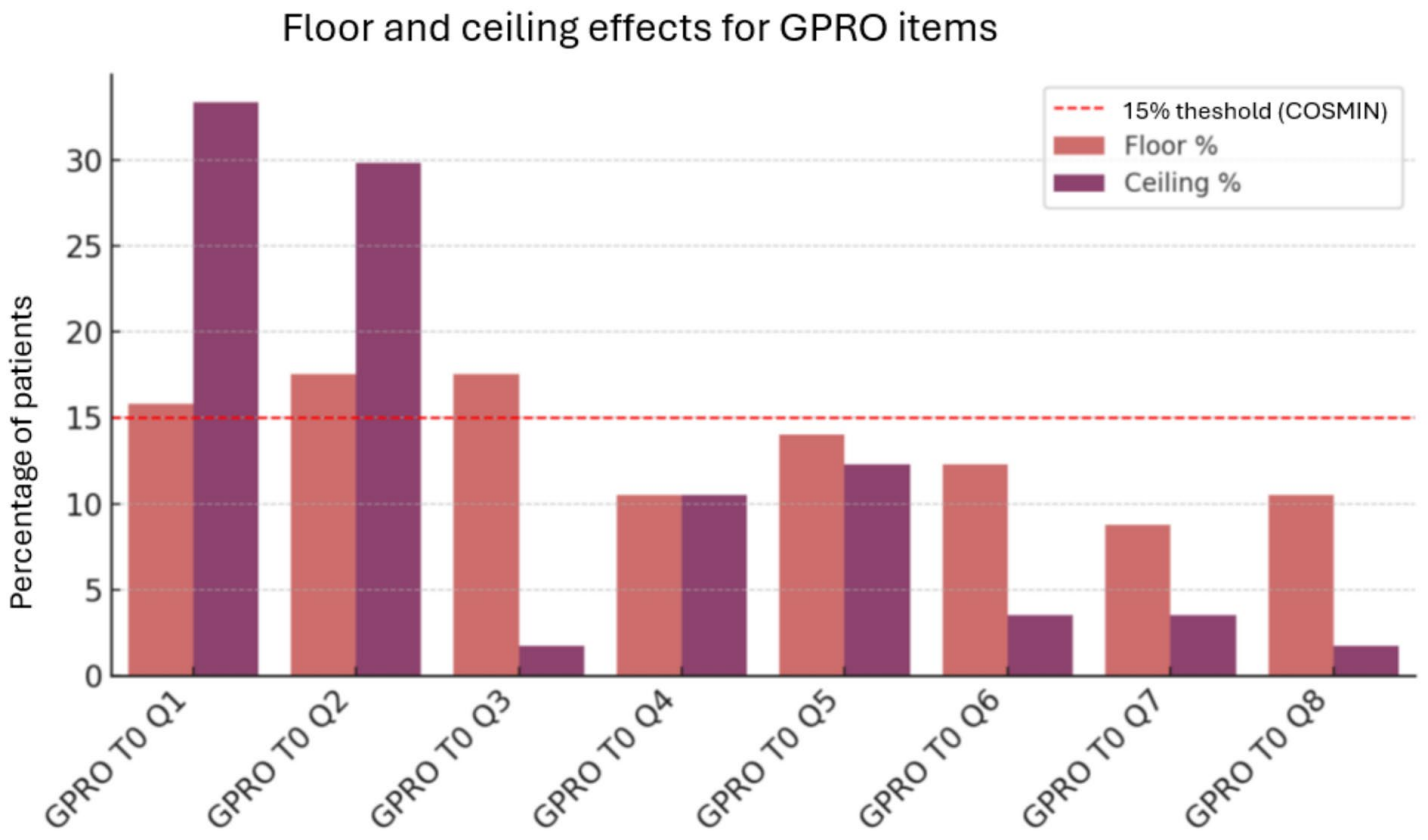
Floor and ceiling effects for GPRO items. The minimum and maximum response rates for each GPRO item suggest that, although most items had values below the COSMIN threshold of 15%, some (particularly Q1, Q2, and Q3) showed a high concentration of extreme scores (floor 17%, ceiling 30–33%), suggesting partial saturation of the scale for these items.

Finally, the analysis of floor and ceiling effects showed that two items exceeded the COSMIN threshold of 15% for one or both saturation parameters. Q1 and Q2 exhibited elevated ceiling and floor effects, indicating a concentration of responses at the extremes for these items. The remaining items demonstrated acceptable distribution patterns (Figure 14).

## 4 Discussion

Genital psoriasis in our cohort (*n* = 57) confirmed its substantial clinical, psychosocial, and sexual impact, consistent with prior evidence highlighting the disproportionate burden of genital involvement despite the often limited cutaneous surface area affected. Patients reported a broad constellation of symptoms, including pruritus, burning, pain or discomfort, fissuring, shame, avoidance behaviors, impaired sexual desire or frequency, and anxiety related to intimacy. Such manifestations underscore the multidimensional nature of the condition, where physical symptoms intertwine with emotional, relational, and functional impairments. Importantly, currently available PROMs and clinical indices only partially capture this complexity: Generic dermatology questionnaires such as the DLQI tend to underestimate genital-specific impairment, while instruments such as the GPSS focus exclusively on physical symptoms and the GenPs-SFQ primarily targets sexual frequency and avoidance. These limitations justified the development of the GPRO questionnaire as a comprehensive, multidimensional PROM designed specifically for genital psoriasis.

In the longitudinal comparison between baseline (T0) and week 16 (T1), the GPRO questionnaire demonstrated a clear and statistically significant reduction in the total score and in all individual item scores. Although the magnitude of significance varied across items, all domains—physical symptoms, psychosocial discomfort, relational and sexual interference, and impact on daily activities—showed a consistent downward trajectory. This uniform improvement indicates strong sensitivity to clinical change and supports the ability of the GPRO questionnaire to reflect patient-relevant outcomes across multiple dimensions of the disease experience. Of particular relevance, the marked reductions in items related to discomfort during sexual activity, avoidance, and perceived shame suggest not only physical improvement but also a recovery of sexual confidence, relational wellbeing, and body image. These aspects, which are often deeply compromised in genital psoriasis, tend to improve more slowly than cutaneous signs; the GPRO questionnaire’s ability to detect these changes reinforces its clinical value as a patient-centered measure.

Linear regression analyses further supported the validity of the instrument. The GPRO questionnaire consistently showed the strongest and most significant association with the genital Physician’s Global Assessment (gPGA) at both T0 and T1, with coefficients of determination exceeding 50%. These findings demonstrate that the GPRO questionnaire aligns closely with objective clinical severity while simultaneously integrating the patient’s subjective perception of burden. The downward shift of the regression line at follow-up, observed at the same GPRO scores, reflects the expected clinical improvement following systemic therapy and supports the responsiveness of the instrument. In contrast, the GPSS showed only moderate correlations with the gPGA, which increased slightly at T1, suggesting that residual physical symptoms become more prominent determinants of clinical severity as the disease improves. The SFQ exhibited a marked decline in its association with the gPGA from baseline to follow-up, highlighting the well-known dissociation between cutaneous remission and recovery of sexual function. Shame, avoidance, fear of rejection, and long-standing negative sexual experiences often persist beyond clinical improvement, and the SFQ captures this prolongation of relational and affective distress.

All PROMs demonstrated very weak and non-significant correlations with the PASI, confirming the anatomical and conceptual specificity of genital PROMs and emphasizing that genital-related distress is largely independent of global skin severity. This observation is fully consistent with the literature, which has repeatedly shown that genital psoriasis exerts a disproportionate psychosocial burden irrespective of overall disease extent.

Internal consistency analyses supported the psychometric robustness of the GPRO questionnaire. Cronbach’s alpha was 0.94, far exceeding the conventional threshold for acceptability and indicating excellent homogeneity among items. The high mean inter-item correlation (0.71) and corrected item–total correlations (all > 0.70) confirm that each item contributes substantially to the underlying construct without redundancy. The “alpha if item deleted” analysis further demonstrated that removing any individual item did not improve reliability, supporting the necessity and relevance of all questions for capturing the multifaceted impact of genital psoriasis.

Responsiveness analyses showed that the GPRO questionnaire captured clinical change over time. The mean GPRO total score decreased from 18.7 at baseline to 5.3 at week 16, corresponding to a mean reduction of 13.4 points in the overall cohort. Moreover, patients classified as responders on the basis of the clinical anchor (ΔgPGA ≤ − 1) showed a significantly greater reduction in GPRO scores than non-responders, and the change in GPRO scores correlated moderately with the change in the gPGA (*r* = 0.52; *p* = 0.003), supporting the responsiveness of the instrument. Anchor-based analyses confirmed these findings: Responders (defined by ΔgPGA ≤ − 1) exhibited significantly greater reductions in GPRO scores than non-responders (*p* < 0.001). The ROC curve yielded an AUC of 0.81 (95% CI 0.72–0.90), demonstrating excellent discriminative capacity. The resulting anchor-based MIC of −1.50 points represents a preliminary but clinically meaningful threshold for interpreting meaningful improvement in individual patients. From a practical perspective, a reduction of approximately 1.5 points in GPRO scores may correspond to the earliest patient-perceivable improvement in genital psoriasis burden, potentially reflecting reduced discomfort, decreased embarrassment or avoidance behaviors, or improved tolerance of intimacy-related situations.

EFA offers important insights into the internal structure of the GPRO questionnaire. The questionnaire was conceptually developed to represent three interrelated domains: (1) physical symptomatology (items Q2–Q4), (2) psychosocial and psychosexual discomfort (items Q1, Q5, and Q6), and (3) impairment in daily activities (items Q7 and Q8). However, when empirically tested at baseline, the EFA revealed the presence of a highly dominant general factor that accounted for approximately 81% of the total variance. All eight items displayed high loadings on this dominant factor (ranging from 0.69 to 0.99), indicating that they all strongly reflect a common latent construct representing the overall impact of genital psoriasis on patients’ lives.

The additional extracted factors showed much weaker loadings and did not support a robust separation into three empirically distinct dimensions in this sample. Communalities were uniformly high (0.52–1.19), confirming that all items are well explained by the factor structure and contribute meaningfully to the core construct. This pattern aligns with what is often observed in PROMs assessing tightly interconnected somatic and psychosocial conditions: The lived experience of genital psoriasis integrates physical, emotional, relational, and functional elements in a unified manner. Consequently, while the theoretical framework of three domains remains clinically meaningful—and may still guide sub-analyses or descriptive interpretation—the empirical evidence supports the use of a single total score for primary clinical evaluation and research.

Construct validity was reinforced by the correlations with external measures. The strong association with the gPGA and the moderate association with the GPSS align with the physical and clinical aspects of the disease, whereas the strong correlation with the avoidance component of the SFQ captures the relational and sexual dimensions. The absence of a correlation with GenPs frequency (Part 1 of the SFQ) suggests that sexual frequency alone is not sensitive to the full spectrum of genital psoriasis distress, supporting the broader scope of the GPRO questionnaire. The examination of score distributions further showed minimal floor or ceiling effects for most items, indicating good discriminative capacity across different levels of disease severity. Only a few items showed mild extremes post-treatment, likely due to true clinical improvement rather than structural limitations of the instrument. These findings do not invalidate the scale; rather, they indicate that individual items may show limited variability in certain clinical contexts and warrant consideration in future refinement studies.

These findings collectively highlight several strengths of the GPRO questionnaire. It is the first PROM designed specifically for genital psoriasis and, therefore, captures aspects that generic dermatological or sexual health instruments systematically miss. Its multidimensional yet empirically unified structure integrates physical, emotional, relational, and functional domains. The instrument demonstrated excellent sensitivity to clinical change, strong internal consistency, coherent construct validity, and practical applicability given its brevity and ease of administration.

Overall, the present findings position the GPRO questionnaire as a valid, reliable, responsive, and clinically meaningful instrument for assessing genital psoriasis, capturing both the physical dimension of disease and its profound psychosocial and relational consequences. Its use as a unified total score appears justified by the empirical evidence, while its conceptual multidimensionality provides a clinically useful framework for interpretation and patient counseling.

## 5 Study limitations

Although this pilot study provides robust preliminary evidence of the internal consistency, convergent validity, and short-term responsiveness of the GPRO questionnaire, several COSMIN-recommended evaluations require a larger sample size and, ideally, a multicenter design to achieve full and comprehensive validation.

According to COSMIN standards, the psychometric validation of a PROM should address three core domains—reliability, validity, and responsiveness. In the present study, some of these assessments could not yet be completed due to methodological and sample size constraints.

Test–retest reliability, which is essential for evaluating the temporal stability of the instrument under clinically unchanged conditions, was not performed. This analysis requires a subgroup of patients with stable disease over a short interval (7–14 days). A minimum of 30 participants is recommended; however, samples of approximately 50 individuals provide more precise estimates of the intraclass correlation coefficient, standard error of measurement, and minimal detectable change.

A further limitation of the present study is the absence of confirmatory factor analysis. Although EFA is appropriate in the early phases of instrument development, the relatively small and monocentric sample precluded the application of more robust confirmatory modeling techniques. As a result, the factor structure identified should be interpreted as preliminary. Future multicenter studies with larger and more diverse populations are warranted to perform CFA, confirm the dimensionality of the GPRO questionnaire, and ensure the stability and cross-cultural validity of the instrument.

Further evaluation of criterion and discriminant validity is warranted. Correlations with clinical severity indices (e.g., the gPGA and PASI) and established PROMs (the GPSS and GenPS-SFQ) would strengthen criterion validity, while known-groups analyses or multivariate models could assess discriminant validity. These analyses generally require samples of ≥100 patients overall and at least 20 participants per comparison group. A multicenter design would also increase representativeness across the diverse phenotypes and severity levels of genital psoriasis. Although responsiveness was assessed over a 16-week interval, COSMIN guidelines highlight the relevance of long-term responsiveness (≥6–12 months) to evaluate the stability and sensitivity of change over time. An extended follow-up at 32 and 46 weeks for a subset of patients is planned, which will also support the estimation of the MIC using an anchor-based approach (e.g., changes in the gPGA).

Finally, full external validation of the GPRO questionnaire will require replication in an independent multicenter cohort of >300 patients. Such a study would allow confirmation of the psychometric properties observed here and assessment of cross-cultural reproducibility and generalizability across different clinical settings.

## 6 Conclusion

This pilot study enabled a preliminary evaluation of the psychometric and clinical properties of the GPRO questionnaire, a new self-reported tool specifically developed to assess the physical, relational, and sexual impact of genital psoriasis. The findings indicate that the GPRO questionnaire can detect significant changes between T0 and T1 across most items, demonstrating good sensitivity to change (responsiveness) and clear alignment with objective clinical improvement as measured by the gPGA. Overall, these results support the preliminary validity and clinical relevance of the GPRO questionnaire as a genital psoriasis-specific tool that captures physical, psychological, and relational dimensions often overlooked by generic questionnaires. Nevertheless, this is a pilot study that provides reliable preliminary results and underscores the need for external multicenter validation to strengthen its applicability in both clinical practice and research.

## Data Availability

The raw data supporting the conclusions of this article will be made available by the authors, without undue reservation.
