# Supplementary material for: Pilot validation of the genital psoriasis reported outcomes questionnaire in an Italian cohort
Source: Front Med (Lausanne). 2026 Jul 29;13:1815709. doi: 10.3389/fmed.2026.1815709 (PMC13461462; doi:10.3389/fmed.2026.1815709)
Supplement: Supplementary file 1 [file Supplementary_file_1.docx]

**Genital Psoriasis Reported Outcome (GPRO)**

**Epidemiological Data**

Sex:

a) Male
b) Female

Age (years):

<24;

b) 25–30;

c) 31–35;

d) 36–45;

e) 46–55;

f) 56–65; g) 66–75;

h) >76

How long have you had psoriasis?

a) Less than 3 months;
b) About 1 year;
c) 3 years or less;
d) 6 years or less;
e) More than 6 years

How long have you had genital psoriasis?

a) Less than 3 months;
b) About 1 year;
c) 3 years or less;
d) 6 years or less;
e) More than 6 years

How long have you been receiving topical and/or systemic treatment for genital psoriasis?

a) Less than 3 months;
b) About 1 year;
c) 3 years or less;
d) 6 years or less;
e) More than 6 years

Do you have a steady partner?

a) No;
b) Yes, for less than 3 months;
c) Yes, for about 1 year;
d) Yes, for 3 years or less;
e) Yes, for 6 years or less;
f) Yes, for more than 6 years

**Disease Activity** (scores assigned to each answer are shown in brackets and are not included in the patient-completed version)

1) Have you felt ashamed in front of your sexual partner(s) during the last 15 days? If yes, since when?
a) Yes, only after the onset of genital psoriasis (also select if other forms of psoriasis appeared simultaneously) (3);
b) Yes, only after the onset of non-genital psoriasis, but worsened by the development of genital psoriasis (2);
c) Yes, even before any form of psoriasis, but worsened after genital psoriasis appeared (1);
d) Yes, even before the onset of genital psoriasis and unchanged after its appearance (0);
e) No (0)
2) Have you experienced pain and/or discomfort and/or burning during sexual intercourse in the last 15 days?
a) Yes, always (3);
b) Yes, about half of the time (2);
c) Rarely (1);
d) No (0)
3) If you answered yes to the previous question, indicate which symptoms were present. Multiple answers may be selected (scores are cumulative).
a) Burning (0.5);
b) Pain (0.5);
c) Itching (0.5);
d) Stinging sensation (0.5);
e) Dryness sensation (0.5);
4) Have you experienced pain and/or discomfort and/or burning in the genital area outside of sexual intercourse during the last 15 days?
a) Always (4);
b) Very often (3);
c) Sometimes in everyday life (2);
d) Only in specific situations (1);
e) Never (0)
5) Have you felt less sexually attractive to your sexual partner during the last 15 days?
a) Yes, only after the onset of genital psoriasis (also select if other forms of psoriasis appeared simultaneously) (3);
b) Yes, only after the onset of non-genital psoriasis, but worsened by genital psoriasis (2);
c) Yes, even before any form of psoriasis, but worsened after genital psoriasis appeared (1);
d) Yes, even before genital psoriasis and unchanged after its appearance (0);
e) No (0)
6) Have you avoided or wanted to avoid sexual intercourse because of genital psoriasis during the last 15 days?
a) Yes, very frequently (3);
b) Yes, on about half of occasions (2);
c) Rarely (1);
d) Never (0)
7) Have you needed to adapt your choice of clothing because of signs or symptoms of genital psoriasis during the last 15 days?
a) Always (4);
b) Very often (3);
c) Sometimes in everyday life (2);
d) Only in specific situations requiring particular clothing and/or underwear (1);
e) Never (0)
8) How much has your genital psoriasis affected your daily quality of life during the last 15 days?
a) Extremely; my daily habits have been profoundly altered compared with before the onset of genital psoriasis (4).
b) Very much; I can maintain the same habits, although with considerable psychological and/or physical difficulties that reduce their frequency and/or quality (3).
c) Moderately; my habits are not profoundly altered, but are performed with slightly lower frequency and quality because of psychological and/or physical difficulties (2).
d) Slightly; I maintain the same habits as before genital psoriasis, although occasional mild embarrassment/discomfort and/or symptoms remain (1).
e) Not at all; I maintain the same habits as before genital psoriasis, with no shame or discomfort interfering with my routine (0).
